# Supplementary material for: Intestinal Microbiota and Metabolomics Reveal the Role of Auricularia delicate in Regulating Colitis-Associated Colorectal Cancer
Source: Nutrients. 2023 Dec 4;15(23):5011. doi: 10.3390/nu15235011 (PMC10708550; doi:10.3390/nu15235011)
Supplement: Supplementary file 1 [file nutrients-15-05011-s001.zip › nutrients-2710795-supplementary.pdf]

Supplementary material

Table S1 Details of antibodies.

Antibody	Molecular weight	Catalog number	Dilution	Company	Area
FFAR2	50 kDa	19952-1-AP	1:1000	Proteintech	Wuhan, China
β-Arrestin 2	50 kDa	10171-1-AP	1:1000	Proteintech	Wuhan, China
IκBα	35 kDa	ab32518	1:4000	Abcam	Cambridge, MA, USA
p-IκBα (Ser32/Ser36)	39 kDa	AF2002	1:1000	Affinity	Cincinnati, OH, USA
TAB1	55 kDa	67020-1-IG	1:5000	Proteintech	Wuhan, China
TAK1	67 kDa	12330-2-AP	1:1000	Proteintech	Wuhan, China
p-TAK1 (Thr184/Thr187)	70 kDa	AF4379	1:1000	Affinity	Cincinnati, OH, USA
IKKα/β	85 kDa	ab178870	1:1000	Abcam	Cambridge, MA, USA
p-IKKα/β (Ser180/Ser181)	87 kDa	AF3013	1:1000	Affinity	Cincinnati, OH, USA
NF-κB p65/RelA	60 kDa	A18210	1:1000	ABclonal	Wuhan, China
p-NF-κB p65 (Ser536)	65 kDa	AF2006	1:1000	Affinity	Cincinnati, OH, USA
IL-1β	30 kDa	A16288	1:1000	ABclonal	Wuhan, China
IL-6	24 kDa	12912S	1:1000	Cell Signaling Technology	Beverly, MA, USA
IL-1α	20 kDa	A2170	1:1000	ABclonal	Wuhan, China
TNF-α	25 kDa	A0277	1:1000	ABclonal	Wuhan, China
MCP1	26 kDa	66272-1-Ig	1:1000	Proteintech	Wuhan, China
GAPDH	37 kDa	E-AB-20032	1;2000	Elabscience	Wuhan, China
Goat Anti-Rabbit (H+L)	/	E-AB-1003	1;4000	Elabscience	Wuhan, China
Goat Anti-Mouse (H+L)	/	E-AB-1001	1;4000	Elabscience	Wuhan, China

Table S2 Relative abundance of top 20 genera.

Sample	Mean of Ctrl	Mean of Model	Mean of ADL	Mean of ADH
Clostridium	0.004202	0.01461	0.002916	0.001994
Turicibacter	0.000745	0.006197	0.002047	0.003385
Phascolarctobacterium	0.013097	0.014018	0.006649	0.006049
Oscillospira	0.018361	0.040055	0.041611	0.025535
Adlercreutzia	0.002303	0.002173	0.001303	0.001486
Desulfovibrio	0.008533	0.011349	0.012245	0.009161
Mucispirillum	0.000834	0.001717	0.001831	0.001791
Bifidobacterium	0.001513	0.001591	0.000462	0.001888
Roseburia	0.004639	0.001511	0.000454	0.000057
[Ruminococcus]	0.007662	0.007036	0.008931	0.005189
Lactobacillus	0.099375	0.057621	0.031702	0.057311
Ruminococcus	0.004628	0.011089	0.015433	0.016147
Coprococcus	0.023208	0.016967	0.019242	0.012326
Paraprevotella	0.001101	0.002141	0.008284	0.003426
Sutterella	0.003923	0.004417	0.006274	0.006652
Bacteroides	0.004252	0.004971	0.008614	0.010838
Allobaculum	0.041358	0.007517	0.010727	0.015557
Flexispira	0.001899	0.001738	0.00299	0.004929
Parabacteroides	0.000769	0.000841	0.002604	0.002768
Akkermansia	0.002036	0.001084	0.001581	0.002793

Table S3 Significantly different metabolites.

Adduct	Name	Mean of Ctrl	Mean of Model	Mean of 0.45g/kg	Mean of 1.35g/kg
(M-H)-	Orotate	19282.34	39213.20	19932.81	27283.77
(M+H)+	Allantoin	19046.22	26409.58	23434.82	17906.86
M+	Choline	35549.59	41856.17	29123.37	23283.80
(M-H)-	(+-)12-HETE	800438.63	964611.34	899755.80	888147.88
(M-H)-	cis-9-Palmitoleic acid	549657.15	616645.32	545667.01	441609.74
(M-H)-	Nervonic acid	22326.85	24278.77	15080.39	16818.34
(M+H)+	Creatinine	50917.70	58337.68	51521.48	38833.17
(M-H)-	Thymidine	7895.45	19949.32	16114.46	15049.20
(M-H)-	2'-Deoxyuridine	21523.55	42700.72	37930.36	31855.35
(M-H)-	2-Ethyl-2-Hydroxybutyric acid	30571.13	39644.76	38311.58	23152.79
(M-H)-	7Z, 10Z, 13Z, 16Z, 19Z-Docosapentaenoic acid	261366.53	268411.64	204910.19	247925.21
(M+H-H2O)+	DL-Indole-3-lactic acid	47974.53	47582.66	37473.85	27348.72
(M-H)-	Behenic acid	10484.59	9857.15	5039.71	6100.14
(M-H)-	Dihomo-gamma-Linolenic Acid	184990.83	175296.86	133159.36	136644.00
(M-H)-	Ethyl glucuronide	3116.66	7241.27	8389.99	4229.35
(M+H)+	L-Carnitine	1645264.84	1503513.57	1227646.83	793151.79
(M+H)+	Pantothenate	42146.06	42576.09	42524.08	35201.10
(M+CH3CN+H)+	1-Palmitoyllysophosphatidylcholine	62345.50	56843.03	31813.78	47735.45
(M+Na-2H)-	1-Oleoyl-L-alpha-lysophosphatidic acid	49069.27	45624.58	29159.38	41434.13
(M-H2O-H)-	Glycerol 3-phosphate	87002.12	75628.16	61269.38	59063.14
(M+H)+	Taurine	186273.48	188442.61	211033.62	86242.75
(M-H)-	3,4-Dihydroxybenzoate (Protocatechuic acid)	25965.26	16725.51	10280.99	5804.65
(M+H-H2O)+	L-Glutamate	40602.05	33500.89	33988.40	22134.09
M+	alpha-Tocopherol (Vitamin E)	173847.09	161320.69	144436.26	173276.27
(M+K)+	Ile-Asn	21848.69	14682.02	12172.01	14646.87
(M+H)+	Anthranilic acid (Vitamin L1)	109909.56	91434.55	133647.14	72262.15
(M+H)+	1-Palmitoyl-sn-glycero-3-phosphocholine	52426.69	35313.38	69672.19	19681.84
(M+H-H2O)+	1-Aminocyclopropanecarboxylic acid	16108.77	18325.88	27232.87	24930.57
(M-H)-	3-Indolepropionic acid	19264.12	6850.39	7837.07	8644.35

(M+H-H2O)+	Dopamine	11857.67	5984.61	7642.94	5839.46
(M-H)-	Phenol	26535.33	17734.05	22560.08	16790.92
(M+CH3COO+2H)+	Cyclohexylamine	16149.28	7295.55	9055.07	10006.53
(M+H)+	L-Palmitoylcarnitine	23467.00	20233.18	27983.09	22442.88
(M+H)+	Nicotinamide N-oxide	11999.32	7114.54	11521.63	15643.40
M+	1-Methylnicotinamide	27252.88	17201.35	24155.77	23874.91
(M-H)-	Cyclopiazonic Acid	8828.82	4707.96	9773.92	10128.77

Figure List

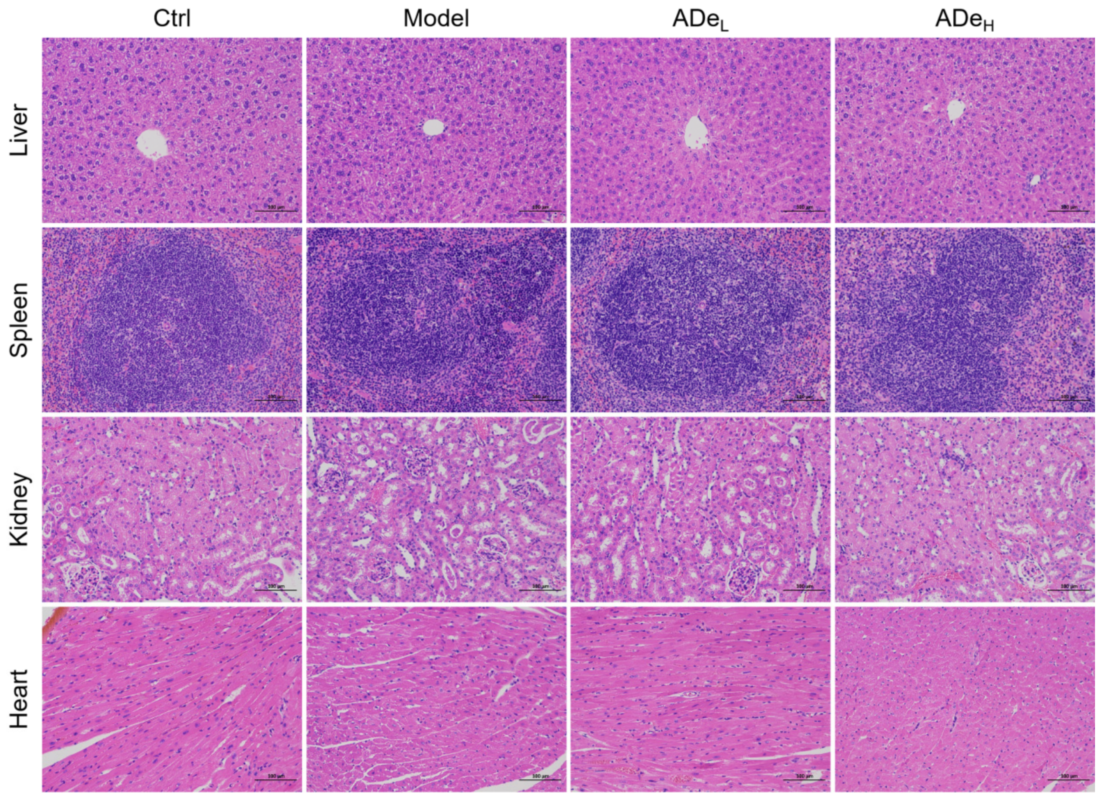

Figure S1 Histopathological analysis of the liver, spleen, kidney, and heart via H&E staining (200×; scale bar: 100 μm).

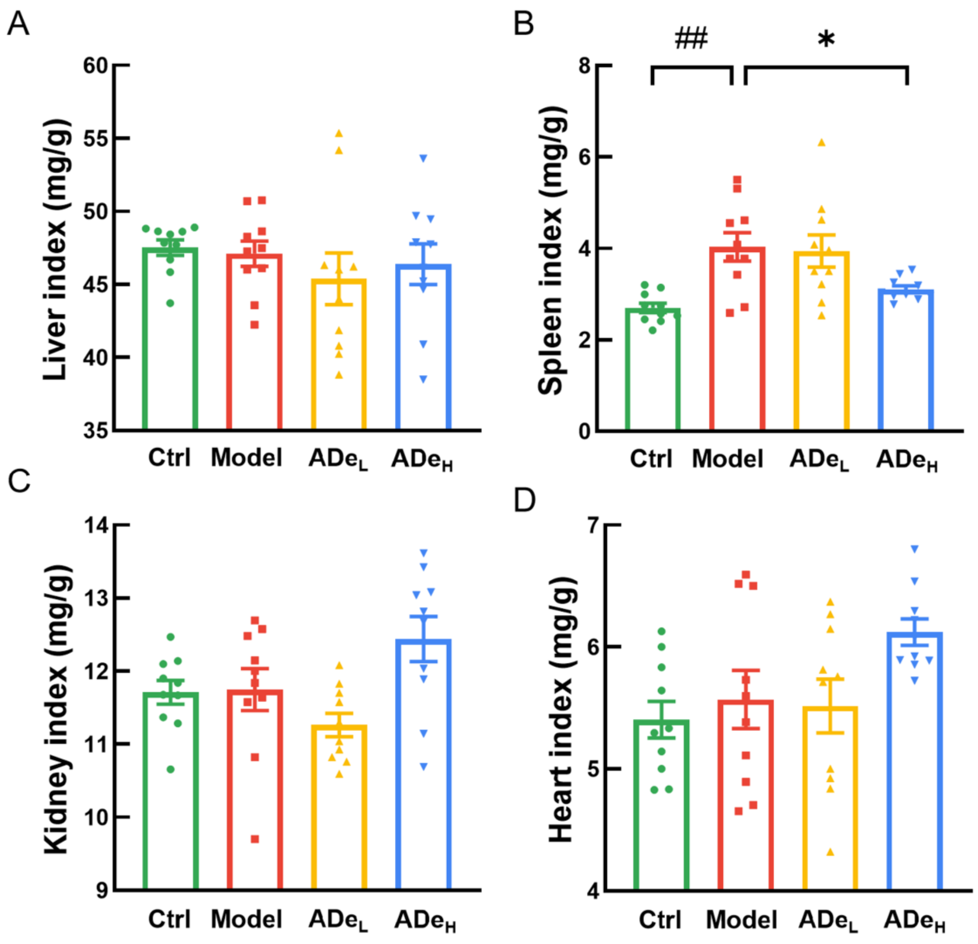

Figure S2 The effect of ADe on organ indices in CAC mice. the (**A**) liver, (**B**) spleen, (**C**) kidney, and (**D**) heart. Data are presented as the means ± S.E.M. Different groups performed with one-way ANOVA followed by a post hoc multiple comparisons (Dunnett) test. $^{##}p < 0.01$ vs. Ctrl group; $^{*}p < 0.05$ vs. model group.
